# Increased Expression of Tim-3 Is Associated With Depletion of NKT Cells In SARS-CoV-2 Infection

**DOI:** 10.3389/fimmu.2022.796682

**Published:** 2022-02-16

**Authors:** Jingzhi Yang, Teding Chang, Liangsheng Tang, Hai Deng, Deng Chen, Jialiu Luo, Han Wu, TingXuan Tang, Cong Zhang, Zhenwen Li, Liming Dong, Xiang-Ping Yang, Zhao-Hui Tang

**Affiliations:** ^1^ Division of Trauma & Surgical Critical Care, Department of Surgery, Tongji Hospital, Wuhan, China; ^2^ Department of Immunology, Tongji Medical College, Huazhong University of Science and Technology, Wuhan, China; ^3^ School of Medicine, Wuhan University of Science and Technology, Wuhan, China

**Keywords:** COVID-19, immunopathogenesis, NKT cells, Tim-3, scRNA-Seq

## Abstract

In the ongoing coronavirus disease 2019 (COVID-19) caused by severe acute respiratory syndrome coronavirus 2 (SARS-CoV-2), natural killer T (NKT) cells act as primary initiators of immune responses. However, a decrease of circulating NKT cells has been observed in COVID-19 different stages, of which the underlying mechanism remains to be elucidated. Here, by performing single-cell RNA sequencing analysis in three large cohorts of COVID-19 patients, we found that increased expression of Tim-3 promotes depletion of NKT cells during the progression stage of COVID-19, which is associated with disease severity and outcome of patients with COVID-19. Tim-3+ NKT cells also expressed high levels of CD147 and CD26, which are potential SARS-CoV-2 spike binding receptors. In the study, Tim-3+ NKT cells showed high enrichment of apoptosis, higher expression levels of mitochondrial genes and caspase genes, with a larger pseudo time value. In addition, Tim-3+ NKT cells in COVID-19 presented a stronger capacity to secrete IFN-γ, IL-4 and IL-10 compared with healthy individuals, they also demonstrated high expression of co-inhibitory receptors such as PD-1, CTLA-4, and LAG-3. Moreover, we found that IL-12 secreted by dendritic cells (DCs) was positively correlated with up-regulated expression of Tim-3 in NKT cells in COVID-19 patients. Overall, this study describes a novel mechanism by which up-regulated Tim-3 expression induced the depletion and dysfunction of NKT cells in COVID-19 patients. These findings not only have possible implications for the prediction of severity and prognosis in COVID-19 but also provide a link between NKT cells and future new therapeutic strategies in SARS-CoV-2 infection.

## Introduction

Coronavirus disease 2019 (COVID-19), a disease caused by severe acute respiratory syndrome coronavirus 2 (SARS-CoV-2), poses a serious threat to public health (1). Up to December 10, 2021, more than 2.6×10^8^ individuals have been infected and more than 5.3×10^6^ patients have died from the disease worldwide [data from Worldometer COVID-19 Data]. Recently, the new Omicron variant of SARS-CoV-2 trigger global panic again.

Dysregulation of the immune system plays a critical role in the COVID-19 pathogenesis. The immune response of SARS-CoV-2 infection is characterized by the differentiation and proliferation of various types of immune cells and the release of immune mediators (2, 3). Natural killer T (NKT) cells, a distinct subset of T cells that express both NK cell markers and T cell markers, are increasingly regarded as cells talented with a hybrid function between NK cells and T cells (4). NKT cells are involved in the host’s defense against virus infection, bridging innate immunity and adaptive immunity (5). NKT cells get activated *via* endogenous lipids presented by CD1d on antigen-presenting cells (APCs), a histocompatibility complex (MHC) class I-like molecule (6), or by stimulating cytokines such as IL-12, IL-18, and type I IFNs (bystander activation) (7, 8). NKT cells exert their antiviral functions by directly lysing target cells, recruiting, stimulating, and regulating other innate cells such as NK cells and neutrophils (9). In addition, NKT cells regulate adaptive cells by promoting B cells to proliferate and produce antibodies, as well as the responses of T cells against intracellular viruses (9). Studies have reported that NKT cells prevent replication of influenza A viruses (IAVs), limit lung damage, and prevent infection of hepatitis virus, dengue virus, and human immunodeficiency virus (HIV) (9–11). Recent studies showed that the number of circulating NKT cells in the peripheral blood of patients with COVID-19 decreased (1, 12–15). NKT cells also showed the ability to enhance vaccine-mediated immune response (16), which may be explained by the fact that NKT cells play distinct roles in different stages of COVID-19. To date, the underlying mechanism of NKT cells depletion and the activity regulation during SARS-CoV-2 infection remain to be further elucidated.

T-cell immunoglobulin and mucin-domain containing-3 (Tim-3) is a type 1 membrane glycoprotein expressed on immune cells including T cells, NKT cells,dendritic cells (DCs) and macrophages, which mediates both innate and adaptive immune responses (17–19). By binding with its natural ligand Galectin-9 (Gal-9), Tim-3 induces T cell apoptosis and exhaustion, thus reducing T cell-mediated immunity and inducing peripheral immune tolerance to viruses including hepatitis B virus (HBV), hepatitis C virus (HCV), dengue virus, influenza virus, herpes simplex virus (HSV) and HIV (18, 20, 21). Recent studies reported that the elevated expression of Tim-3 in skin tissue T cells and peripheral blood mononuclear cells (PBMCs) is associated with deregulation of T cell immune response in SARS-CoV-2 infection (22, 23). In addition, our previous study has shown that Tim-3 plays a pivotal role in the regulation of NKT cell functions during severe bacterial infection (17, 24). However, whether NKT cells are involved in the antiviral immune response against SARS-CoV-2 infection by Tim-3 remains unclear.

In recent study, Zhang et al. performed scRNA-seq analysis of peripheral blood mononuclear cells (PBMCs) from a large cohort of COVID-19 patients, including 81 PBMCs samples from patients with COVID-19 (25). In their study, NKT cells were not classified in cell clusters results. Here, we reanalyzed the data to evaluate the number and function changes of circulating NKT cells. The findings indicated that the number of circulating NKT cells in COVID-19 patients decreased while Tim-3+NKT cells increased, and the expression of Tim-3 was associated with the outcomes of COVID-19 patients. Moreover, the elevated expression level of Tim-3 may be induced by IL-12 produced by DCs/monocytes in SARS-CoV-2 infection. In addition, we validated these results in two other scRNA-seq cohorts and single-cell epitope data. These results indicated that NKT cells are strongly involved in the development of dysregulated immune responses in COVID-19 patients. Tim-3+ NKT cell subsets may be a potential indicator for predicting the outcome of COVID-19, and there is a possibility that the regulation of NKT cells by Tim-3 signal pathway could be a new strategy for immunotherapy in patients with COVID-19.

## Materials and Methods

### Acquisition, Processing, and Integration of Single-Cell RNA Sequencing Datasets

The scRNA-seq datasets in this study were downloaded from Gene Expression Omnibus (GEO) database (GSE158055, GSE168453, and GSE175450). The GSE158055 scRNA-seq dataset was recently published by Zhang et al., including 1462702 cells from 81 advanced COVID-19 patients, 140 recovered COVID-19 patients, and 28 healthy controls, and will be used as a discovery cohort (25). The GSE168453 dataset was constructed by multiplexed single-cell epitope and transcriptome sequencing from PBMCs samples of 54 COVID-19 patients and 11 healthy controls. The GSE175450 scRNA-seq dataset includes PBMCs and T cells samples from 19 advanced COVID-19 patients and 6 healthy controls. The GSE168453 dataset and GSE175450 dataset will be used as validation cohorts. The gene expression matrix was analyzed by R software (v4.0.5) with the Seurat package (v4.0.2) (26). Low-quality cells were discarded according to the following criteria: (1) the number of unique molecular identifiers (UMIs) was less than a quarter of the median of each batch; (2) the number of UMIs exceeds 3 times the median of each batch. And for the GSE168453 dataset, doublet droplets were removed by using the offered Freemuxlet results files. After low-quality cells removal, samples with cells less than 500 were excluded. This study relies entirely on publicly available datasets and hence does not require institutional review board review.

After removal of low quality cells and samples, the gene expression matrix was normalized by the NormalizeData function, and then 2000 features were calculated by the FindVariableFeatures function to select genes with a high intercellular variation. To reduce batch effects between platforms, labs or sample processing, the FindIntegrationAnchors function was used to identify anchors between individual batches based on the previously calculated 2000 features. The selected anchors were input into the IntegrateData function to obtain an integrated gene expression matrix corrected for the batch effect, which was used for subsequent analysis. Then, the ScaleData function and the RunPCA function were performed with the default parameters to diminish the dimensionality of the integrated gene expression matrix. Next, the JackStrawPlot and ElbowPlot functions were conducted to determine the major dimensionality of the dataset, and 50 components were selected. Finally, the FindNeighbors and FindClusters function were used to identify cell clusters, and the RunUMAP function was performed to obtain nonlinear dimensional reduction results.

### Cluster Marker Calculation and Cell Cluster Annotation

The cells were clustered together, and the cell clusters were visualized and projected into two-dimensional space by Uniform manifold approximation and projection (UMAP), resulting in a clear separation between clusters. The differential expression genes (DEGs) between the identified clusters were calculated as markers using the FindAllMarkers function in Seurat. Clusters were annotated based on expressions of canonical gene markers of particular cell types, including CD4+ T cells (*CD3D*+*CD4*+), CD8+ T cells (*CD3D*+*CD8A*+), γδ T cells (*TRGV9*+*TRDV2*+), mucosal-associated invariant T (MAIT) cells (*SLC4A10*+*TRAV1-2*+), NK cells (*KLRF1*+), B cells (*MS4A1*+), plasma B cells (*MZB1*+), CD14+ monocytes (*LYZ*+*CD14*+), CD16+ monocytes (*LYZ*+*FCGR3A*+), monocyte-derived dendritic cells (mono DCs; *CD1C*+), plasmacytoid dendritic cells (pDCs; *LILRA4*+) and platelets (*PPBP*+) (27). Due to the poor knowledge of NKT mRNA markers, NKT cell clusters were classified by three different methods (28). The methods were as follows: The NKT cell test datasets (GSE128243, GSE124731, GSE128626 and GSE28726) were analyzed with SingleR R package (v1.4.1) for annotation (29); Annotation was performed by calculating the expression of NK-associated genes, including *CD16*, *NKP30*, *NKP46*, *2B4*, *NKG2D*, *CD122*, *CD56* and *CD160* (30); Annotation was performed by analyzing the similarity to NK cells based on the results of hierarchical clustering (4). All three classification methods resulted in common annotation, and NKT cell clusters were identified.

### Identification and Functional Enrichment of Differentially Expressed Genes

The differential expression genes (DEGs) between Tim-3+ NKT cells and Tim-3- NKT cells were identified by the FindMarkers in Seurat and Wilcoxon rank-sum test was used to calculate p values. Gene Set Enrichment Analysis (GSEA) on the biological process (BP) of the DEGs was performed by using gseGO in clusterProfiler R package (v.3.18.1) (31).

### Single Cell Pseudotime Trajectory Construction and Analysis

Single cell pseudotime trajectories were constructed by using monocle R package (v2.18.0) (32). In brief, the estimate SizeFactors function in monocle was used to calculate cell specific size factor to normalize the gene expression matrix. Next, the DDRTree algorithm was used to project gene expression matrix into a lower dimensional space based on the 2000 features selected before. Finally, single cells were ordered into a trajectory with branch points by order Cells function in monocle.

### Statistical Analysis

The statistical analysis was performed by R software (v4.0.5). Wilcoxon rank-sum test was used by wilcox.test function to calculate statistical significance and Spearman correlation was used to evaluate correlation coefficient.

## Results

### Single-Cell Transcriptional Profiling of PBMCs in COVID-19

To elucidate the immunological features of COVID-19, the scRNA-seq dataset of PBMCs recently published by Zhang et al. (GSE158055) was reanalyzed as a discovery cohort, including 81 PBMCs samples of patients with COVID-19 in progression, 140 PBMCs samples of patients with COVID-19 in convalescence, and 28 PBMCs samples of healthy donors. After data filtering, samples with cells less than 500 were excluded. Finally, 28 samples of healthy controls, 73 samples of COVID-19 patients in progression, and 129 samples of COVID-19 patients in convalescence remained. The 73 samples of patients in progression were categorized into three groups according to their clinical conditions: patients with mild or moderate COVID-19 (n=24), discharged patients with severe or critical COVID-19 (n=36), and deceased patients with severe or critical COVID-19 (n=13) (**Table 1**). And the 129 samples of patients in convalescence were categorized into two groups: patients after mild or moderate COVID-19 (n=79) and patients after severe or critical COVID-19 (n=50) (**Table 1**). After the quality-control process, 1333525 single cells were obtained with an average of 4581 unique molecular identifiers (UMIs) and 27943 genes represented.

**Table 1:** Information of single-cell datasets.

Dataset		GSE158055	GSE168453	GSE175450
Group	healthy	82	11	12
	mild	24	N/A	12
	moderate		23	N/A
	severe	36	23	17
	critical		23	N/A
	died	13	N/A	N/A
	mild recover	79	N/A	12
	severe recover	50	N/A	10
Cell count		1333525	729812	178325
Country		China	USA	Germany
Sample		PBMC & T/B cells	PBMC	PBMC & T cells
Cohort		Discovery	Validation	Validation

N/A, not available.

**Figure 1:**
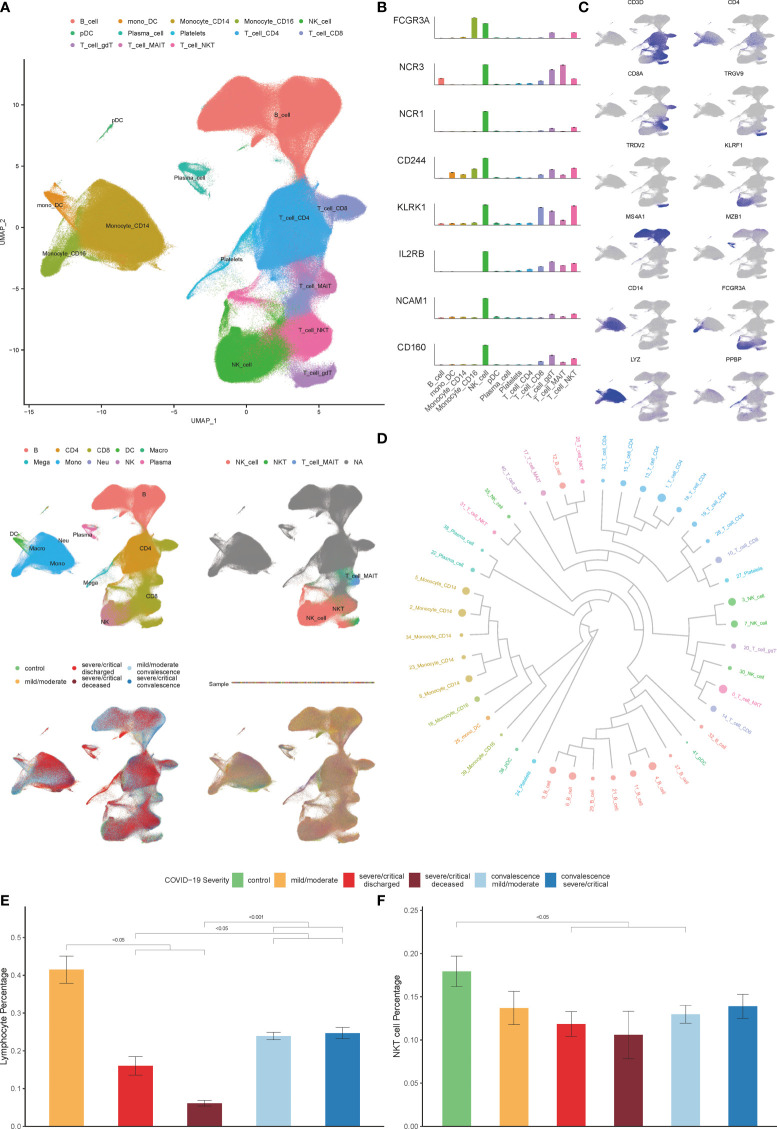
Single-cell transcriptional profiling of PBMCs in COVID-19. **(A)** Integration analysis results of patients with COVID-19 and controls showing UMAP visualization, including integration analysis result of 13 major cell clusters annotated (top), the cell clustering results in origin data (middle left), the result of NKT cell clusters annotated by SingleR (middle right), the result of patients with COVID-19 and healthy controls (bottom left), the result of PBMCs samples with no batch effect observed (bottom right). **(B)** Expression of NK-associated genes, including *CD16* (*FCGR3A*), *NKP30* (*NCR3*), *NKP46* (*NCR1*), *2B4* (*CD244*), *NKG2D* (*KLRK1*), *CD122* (*IL2RB*), *CD56* (*NCAM1*) and *CD160* in cell clusters. **(C)** Known cell markers used to identify PBMCs cell types. **(D)** The result of cell clusters showing by dendrogram **(E)** Percentage of lymphocytes in COVID-19 patients derived by the routine blood test. **(F)** Percentage of NKT cells in COVID-19 patients and controls derived by single cell datasets. (Wilcoxon test).

Thirteen clusters of PBMCs were identified by using unsupervised hierarchical clustering and visualization with uniform manifold approximation and projection (UMAP) (**Figure 1A**). Twelve types of major cell were annotated by expressions of canonical gene markers, which included CD4+ T cells (*CD3D*+*CD4*+), CD8+ T cells (*CD3D*+*CD8A*+), γδ T cells (*TRGV9*+*TRDV2*+), mucosal-associated invariant T (MAIT) cells (*SLC4A10*+*TRAV1-2*+), NK cells (KLRF1+), B cells (*MS4A1*+), plasma B cells (*MZB1*+), CD14+ monocytes (*LYZ*+*CD14*+), CD16+ monocytes (*LYZ*+*FCGR3A*+), monocyte-derived dendritic cells (mono DCs; *CD1C*+), plasmacytoid dendritic cells (pDCs; *LILRA4*+) and platelets (*PPBP*+) (**Figure 1C**) (27). Various experiments were designed for the classification of NKT cells, and the three subsets of NKT cells include type I NKT cells, type II NKT cells, and NKT–like cells (4). NKT cell type was annotated by analyzing NKT cell test datasets (GSE128243, GSE124731, GSE128626 and GSE28726) with SingleR R package (29); Annotation was performed by calculating the expressions of NK-associated genes, including *CD16*, *NKP30*, *NKP46, 2B4*, *NKG2D*, *CD122*, *CD56* and *CD160* (30); Annotation was performed by analyzing the similarity to NK cells based on the results of hierarchical clustering (4). As expected, highly consistent results of NKT cells annotation were obtained. Therefore, all 13 major cell types were classified, which could be divided into 55 cell clusters by Seurat R package at a 1.5 resolution (**Figures 1B, D**).

As shown in **Figure 1E**, the decrease in the percentage of lymphocytes in patients is associated with the severity of COVID-19, consistent with previous reports (1, 12, 33, 34). We further explored the effect of COVID-19 on the composition of immune cells in PBMCs according to the scRNA-seq data analysis. Consistent with previous studies, we found that the percentage of CD8+T cells, MAIT cells, γδ T cells, mono-DCs, and pDCs decreased significantly as the disease progressed, while the percentage of plasma B cells, CD14+ monocytes, and platelets increased significantly, however, there was no significant change in the number of NK cells (**Figure S1**) (25, 27, 35).Of note, the percentage of NKT cells in severe COVID-19 patients decreased significantly in both progression and convalescence (**Figure 1F**), which is consistent with previous studies (13, 14).

### The Expression Level of Potential SARS-CoV-2 Spike Binding Receptors in NKT Cells

**Figure 2:**
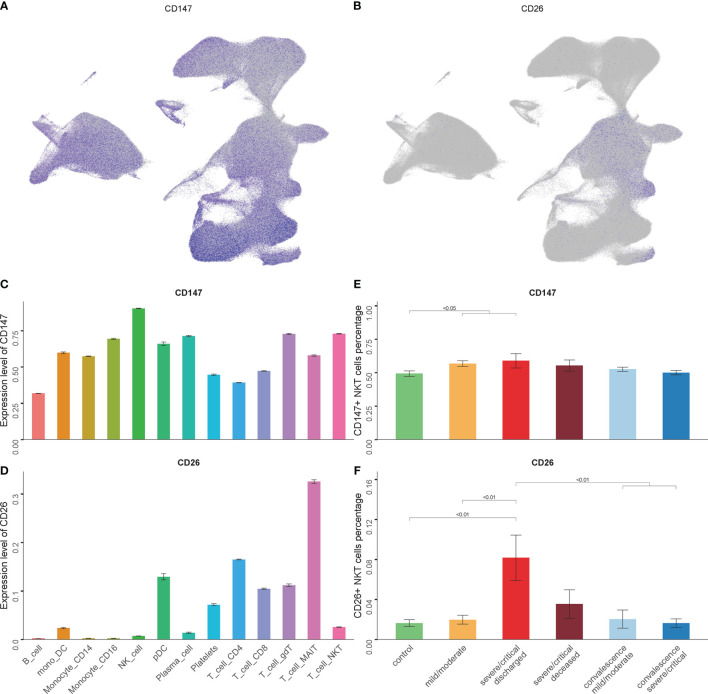
The expression levels of CD147 and CD26 in NKT cells. **(A, B)** Expression of CD147 and CD26 in PBMCs showing UMAP visualization. **(C, D)** Expression of CD147 and CD26 in PBMC subsets. **(E, F)** Percentage of CD147+ NKT cells and CD26+ NKT cells in COVID-19 patients and controls. (Wilcoxon test).

ACE2 is one of the most important receptors on the host cells mediating SARS-CoV-2 infection by binding to the spike protein (36). In the present study, none of the analyzed NKT cells expressed ACE2, though the scRNA-seq data showed few ACE2 expressed in PBMCs (data not shown). Recently, several other receptors have been reported to involve in mediating SARS-CoV-2 infection for host cells, including CD147 and CD26 (37, 38). The expression levels of CD147 and CD26 in NKT cells were observed. The findings indicated the highest level of CD147 and CD26 expression detected in NKT cells (**Figures 2A–D**). The relationship between disease severity and CD147+ NKT cells or CD26+ NKT cells was examined during SARS-CoV-2 infection. As shown in **Figure 2E**, a higher proportion of CD147+ NKT cells were revealed among COVID-19 patients. Moreover, the proportion of CD26+ NKT cells significantly increased as the disease progressed and was restored in convalescence (**Figure 2F**).

### Tim-3 Expression on NKT Cells Is Associated With Disease Severity and Outcome of COVID-19 Patients

**Figure 3:**
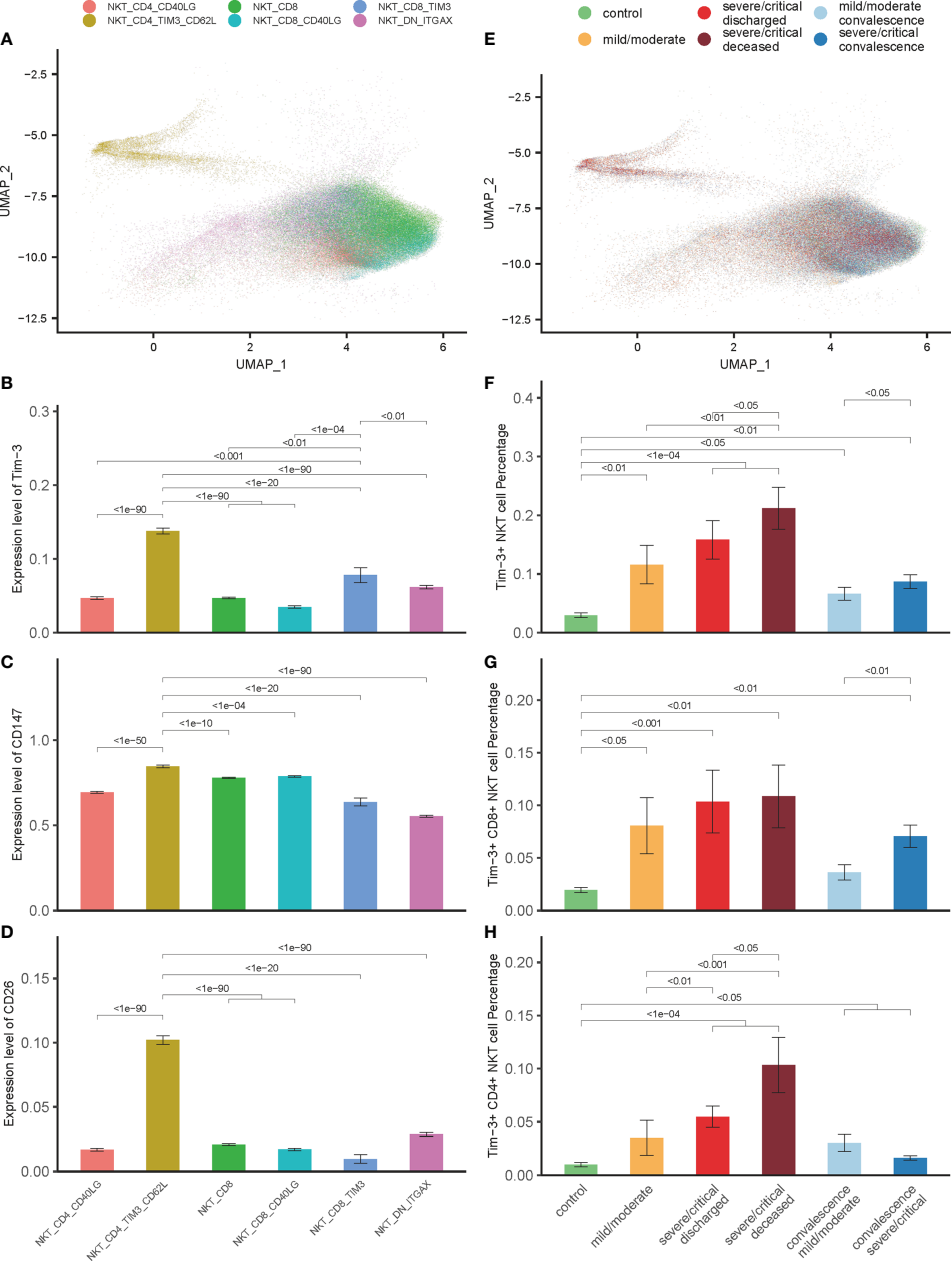
Tim-3 expression in NKT cells associated with disease severity and outcome in COVID-19 patients. **(A)** Integration analysis results of NKT cells in COVID-19 patients and controls with 6 NKT cell clusters. **(B)** Expression of *Tim-3* in 6 NKT cell clusters. **(C, D)** Expression of *CD147* and *CD26* in 6 NKT cell clusters. **(E)** Integration analysis results of NKT cells in COVID-19 patients and controls with no batch effect between samples. **(F–H)** Percentage of Tim-3+ NKT cells, Tim-3+ CD8+ NKT cells and Tim-3+ CD4+ NKT cells in COVID-19 patients and controls. (Wilcoxon test).

For further analysis, NKT cells of 202 COVID-19 patients and 28 controls were grouped into 6 cell subtypes, including NKT_CD4_CD40LG, NKT_CD4_TIM3_CD62L, NKT_CD8, NKT_CD8_CD40LG, NKT_CD8_TIM3, and NKT_DN_ITGAX (**Figure 3A**). Our previous studies have demonstrated that the Tim-3 signal plays an essential role in mediating the impaired function of NKT cells in mice model of polymicrobial intra-abdominal infection (17, 24, 39). In the current study, the role of NKT cells expressing Tim-3 was examined in the pathogenesis of COVID-19. The 6 NKT cells subsets were analyzed, and Tim-3 expression was elevated in 2 NKT subsets—NKT_CD4_TIM3_CD62L and NKT_CD8_TIM3 (**Figure 3B**). Although the total number of NKT cells decreased in the COVID-19 progression phase as shown in **Figure 1E**, the number of Tim-3+ NKT cells increased significantly (**Figures 3F–H**). In addition, this elevation could be detected in all patients with varying severity (**Figure 3E**), and the more severe the COVID-19 was, the greater the number of Tim-3+ NKT cells was detected (**Figures 3F–H**). It was also true for COVID-19 patients in convalescence that the number of Tim-3+ NKT cells was elevated compared to controls (**Figure 3F**). This elevation was unique in Tim-3+ NKT cells, since the other NKT cell subsets, including NKT_CD4_CD40LG, NKT_CD8, NKT_CD8_CD40LG, and NKT_DN_ITGAX, did not show a similar change (**Figure S2**). The relationship between CD147, CD26 and Tim-3 was observed in the 6 isolated NKT cell subsets, and Tim-3+ NKT cells were accompanied with high expression levels of CD147 and CD26, especially in the NKT_CD4_TIM3_CD62L subset (**Figures 3C, D**). Tim-3+ NKT cells have a trend to express more CD147 and CD26 in COVID-19 patients, which might suggest Tim-3+ NKT subset cells are highly susceptible to SARS-CoV-2 infection.

### Tim-3 Expression Is Associated With NKT Cells Apoptosis in COVID-19 Patients

**Figure 4:**
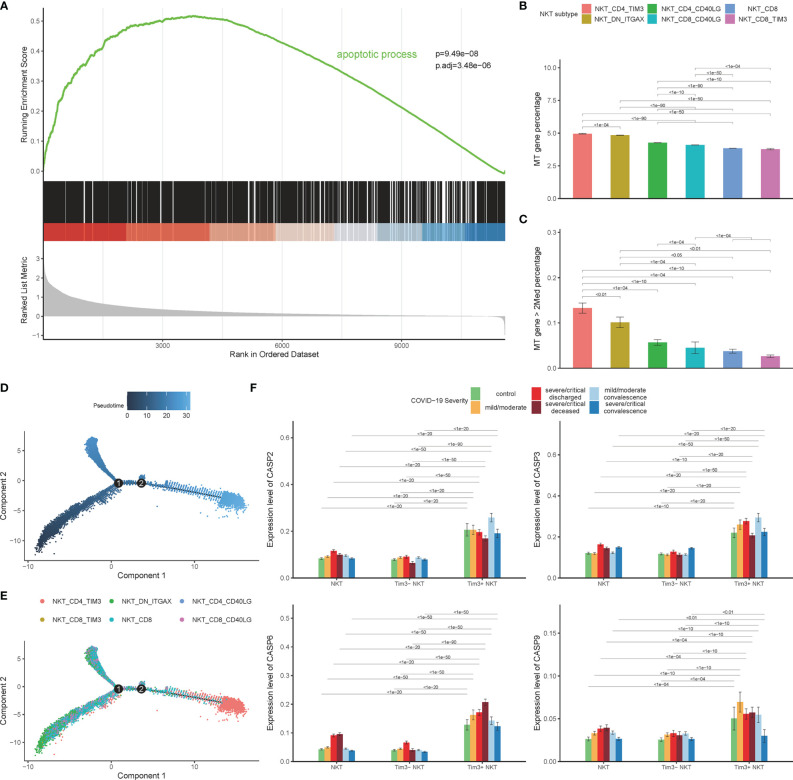
Tim-3 expression associated with NKT cells apoptosis in COVID-19. **(A)** Gene set enrichment analysis (GSEA) result of apoptotic process pathway. **(B)** Expression of mitochondrial genes on 6 NKT cell clusters. **(C)** Percentage of mitochondrial genes highly expressed NKT cells on 6 NKT cell clusters. **(D, E)** Pseudo-time analysis results. **(F)** Expressions of caspase genes on Tim-3+ NKT cells and Tim-3- NKT cells in COVID-19 patients and controls. (Wilcoxon test).

Our recent study has demonstrated that Tim-3 is a negative regulator of cell response through promoting NKT cells apoptosis in mice model of polymicrobial intra-abdominal infection (39). The relationship between the up-regulated expression of Tim-3 in NKT cells and depletion of NKT cells during SARS-CoV-2 infection was firstly observed. Gene Set Enrichment Analysis (GSEA) was performed to unfold the potential biological processes related to Tim-3 in COVID-19 patients. GSEA analysis showed that the apoptotic process pathway was significantly enriched, with an adjusted p value 3.48×10^-5^ (**Figure 4A**). Releasing of mitochondrial (mt) genes into the cytoplasm is associated with cell apoptosis (40), and a large proportion of mt genes are unfavorable to cell development (41). Mt genes in lymphocytes account for about 5% of the total mRNA content (42). The expression of mt genes in Tim-3+ NKT cells was significantly higher than that of other NKT cells, consistent with the results of GSEA (**Figure 4B**). Tim-3+ NKT cells account for the highest proportion in the high-mt-gene-expressed NKT cells subset, which is defined as more than twice the average percentage of mt genes (**Figure 4C**). Subsequently, to study molecular characteristics of NKT cells during the disease, all NKT cells were ordered in pseudo time to reconstruct the trajectory of NKT maturation by using monocle R package. The results of the pseudo time analysis showed that the Tim-3+ NKT cells were positioned at the end of the tree, indicating that Tim-3+ NKT cells were in a late stage of maturation (**Figures 4D, E**).

To shed light on the process of Tim-3+ NKT cell apoptosis, we investigated the expression levels of caspase genes in all COVID-19 patients and healthy controls. Consistent with the apoptosis trend of Tim-3+ NKT cells, an elevated expression level of almost all caspase genes in Tim-3+ NKT cells was observed in controls and patients with mild and severe COVID-19, especially caspase-2, caspase-3, caspase-6, and caspase-9 (**Figure 4F**).

### Tim-3 Expression and Functional Status of NKT Cells in COVID-19

**Figure 5:**
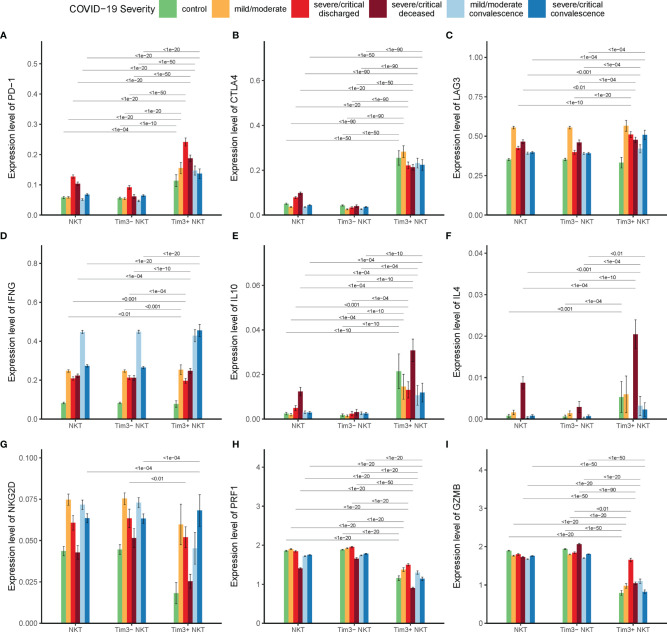
Tim-3 expression and functional status of NKT cells in COVID-19. **(A–C)** Expressions of co-inhibitory receptor genes *PD-1*, *CTLA4*, and *LAG3* on Tim-3+ NKT cells and Tim-3- NKT cells in COVID-19 patients and controls. **(D–F)** Expressions of cytokine genes *IFN-γ*, *IL-10*, and *IL-4* on Tim-3+ NKT cells and Tim-3- NKT cells in COVID-19 patients and controls. **(G–I)** Expressions of cytotoxicity related genes *NKG2D*, *PRF1*, and *GZMB* on Tim-3+ NKT cells and Tim-3- NKT cells in COVID-19 patients and controls. (Wilcoxon test).

The function of co-inhibitory receptors, including Tim-3, programmed death-1 (PD-1), cytotoxic T lymphocyte antigen-4 (CTLA-4), and lymphocyte-activation gene 3 (LAG-3), are critical for lymphocyte homeostasis prompting it a novel target for treatment in tumor and infection (43, 44). It is important to investigate the expression levels of these co-inhibitory receptors in Tim-3+ NKT cells. Our results showed that the expression of these co-inhibitory receptors in Tim-3+ NKT cells was significantly higher than that in TIM-3-NKT cells (**Figures 5A–C**).

Unlike conventional T cells, the most important characteristic of NKT cells in infection is the rapid activation and substantial production of cytokines such as IFN-γ, IL-10, and IL-4. To find out the relationship between the up-regulated expression of Tim-3 and cytokine production of NKT cells in COVID-19, the expressions of *IFN-γ*, *IL-10*, and *IL-4* were examined. We found that the expression of *IFN-γ* in Tim-3+ NKT cells was significantly compared with Tim-3- NKT cells in deceased severe/critical group and recovered severe/critical group (**Figure 5D**). To our surprise, we found that the expression of *IL-10* in Tim-3+ NKT cells was significantly higher than Tim-3- NKT cells under every condition (**Figure 5E**). NKT cells expressed few *IL-4* in controls, discharged groups, and recovered groups, but the production of IL-4 peaked in the deceased group. The expression of *IL-4* in Tim-3+ NKT cells was significantly higher than Tim-3- NKT cells in deceased COVID-19 patients (**Figure 5F**).

To find the relationship between the Tim-3 expression and cytotoxicity of NKT cells, the expressions of *NKG2D*, *PRF1* and *GZMB* were assayed. As shown in **Figures 5G-I**, the expression levels of NKG2D, PRF1, and GZMB in Tim-3+ NKT cells were reduced under almost all conditions.

### Correlation Between IL-12 and Expression of Tim-3 on NKT Cells

**Figure 6:**
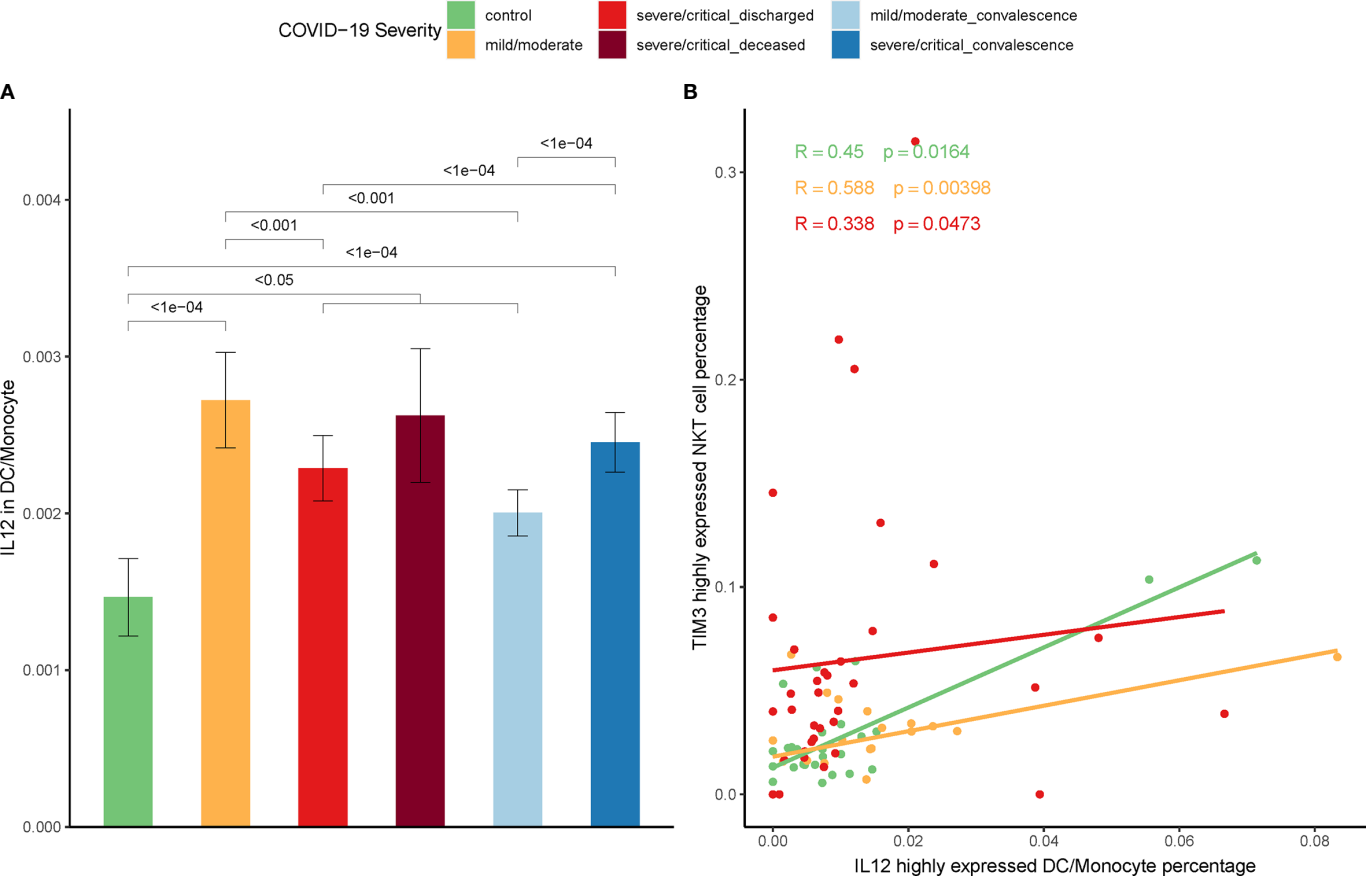
Correlation between IL-12 and expression of Tim-3 on NKT cells. **(A)** Expression of *IL-12* on DCs/monocytes in COVID-19 patients and controls. (Wilcoxon test) **(B)** Correlation between the percentage of *IL-12* highly expressed DCs/monocytes and the percentage of *Tim-3* highly expressed NKT cells in COVID-19 patients (Spearman correlation coefficient).

Our previous work on the mice model indicated that IL-12 secreted by stimulated DCs induced Tim-3 over-expression on NKT cells (39). To ascertain this relationship between IL-12 secretion and Tim-3 over-expression in COVID-19, *IL-12* expression on DCs and monocytes were firstly assayed. In line with the increase expression of Tim-3 in NKT cells in COVID-19 patients, the *IL-12* expression in DCs/monocytes was significantly higher in patients than controls (**Figure 6A**). Additionally, the proportion of DCs/monocytes with high *IL-12* expression was positively correlated with the proportion of NKT cells with high expression of *Tim-3* in discharged COVID-19 patients. The Spearman correlation coefficient R=0.588, p=0.00398 in mild/moderate patients, and R=0.338, p=0.0473 in severe/critical patients was shown in **Figure 6B**.

### Validation With Other Cohorts and Single-Cell Epitope Data

Two cohorts were included as validation cohorts to validate the discovery of Tim-3+ NKT cells in COVID-19 (GSE168453 and GSE175450). The information of each cohort was shown in **Table 1**. Similar data processing, integration, clustering, and annotation were performed on the validation cohorts.

**Figure 7:**
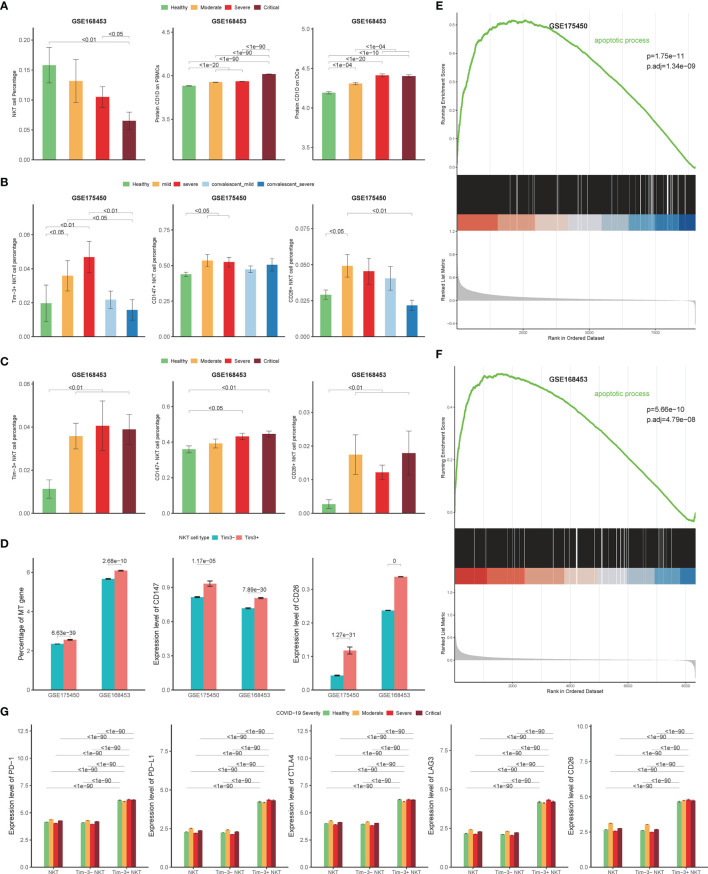
Validation cohort results. **(A)** Percentage of NKT cells in COVID-19 patients and controls derived by scRNA-seq data from GSE168453 (left), protein expression levels of CD1d on PBMCs (middle) and DCs (right) derived by single-cell epitope data from GSE168453. **(B, C)** Percentage of Tim-3+ NKT cells, CD147+ NKT cells, and CD26+ NKT cells in COVID-19 patients and controls derived by scRNA-seq data from validation cohorts. **(D)** Expression of mitochondrial genes, *CD147*, and *CD26* in Tim-3+ NKT cells and Tim-3- NKT cells in validation cohorts. **(E, F)** Gene set enrichment analysis (GSEA) results of apoptotic process pathway in validation cohorts. **(G)** Protein expression levels of PD-1, PD-L1, CTLA4, LAG3, and CD26 on Tim-3+ NKT cells and Tim-3- NKT cells in COVID-19 patients and controls derived by single-cell epitope data from GSE168453. (Wilcoxon test).

As expected, the NKT cells proportion was decreased significantly in severe COVID-19 patients in the GSE168453 cohort (GSE175450 cohort not suitable for calculating NKT cells proportion) (**Figure 7A**). Several viruses, such as Kaposi’s sarcoma-associated herpesvirus, HSV and HIV, can use different mechanisms to reduce CD1d expression, which may lead to impairment of NKT cells (6). To check whether the decrease of circulating NKT cells is related to CD1d deficiency in COVID-19, CD1d protein expression levels on PBMCs and DCs were assayed through single-cell epitope data of GSE168453. As shown in **Figure 7A**, CD1d protein expression levels did not decrease, but increased as the disease progressed, on both PBMCs and DCs. The evidence, therefore, did not support the hypothesis that the decrease of NKT cells in COVID-19 was related to CD1d deficiency.

In addition, the number of Tim-3+ NKT cells increased in both validation cohorts as the disease progressed, with statistical significance (**Figures 7B, C**). And the proportion of Tim-3+ NKT cells recovered as well in convalescence phase, consistent with the discovery cohort (**Figure 7B**). The Tim-3+ NKT cells also showed more mt gene detected than Tim-3- NKT cells (**Figure 7D**). GSEA results were consistent with the discovery cohort, showing that the apoptotic process pathway was enriched in Tim-3+ NKT cells, with adjusted p-value 1.34×10^-9^ in GSE175450 and adjusted p-value 1.48×10^-7^ in GSE168453 (**Figures 7E, F**).

Consistent with the discovery cohort, COVID-19 patients showed a higher proportion of CD147+ NKT cells and CD26+ NKT cells than controls (**Figures 7B, C**). And Tim-3+ NKT cells subset had a trend to express more CD147 and CD26 than Tim-3- NKT cells subset (**Figure 7D**).

To verify the results of the functional status of Tim-3+ NKT cells derived from the discovery cohort, single-cell epitope data from GSE168453 was used. Consistent with the discovery cohort result, the protein expression levels of PD-1, PD-L1, CLTA4, LAG3, and CD26 were significantly higher in Tim-3+ NKT cells than Tim-3- NKT cells under every condition (**Figure 7G**).

## Discussion

NKT cells play an important role in virus infection. After activation by TCR binding to CD1d directly, or by NKT cell-stimulating cytokines such as IL-12, IL-18, and type I IFNs, NKT cells devote to the host’s defense against virus infection by secreting pro- inflammatory or anti- inflammatory cytokines to regulate the activation, recruitment, and differentiation of other immune cells. The effects are critical for the elimination of pathogens and can be inhibited by apoptosis of NKT cells caused by immunosuppression during severe virus infection (45). It was reported that *Cd1d* ^-/-^ mice, which lacked CD1d-dependent NKT activation, were more susceptible to HSV infection, with more severe disease, greater spread of the virus to spinal ganglia, and delayed clearance of virus (46). Impairment of NKT cells may also lead to uncontrolled virus infection in humans (6).

COVID-19 is an acute viral disease caused by SARS-CoV-2 infection, and NKT cells have been reported to play an important role in COVID-19 different stages, including silent SARS-CoV-2 infection stage (47), progressing stage (48), convalescence stage (15), and vaccination (16). However, little is known about the regulatory mechanisms of NKT cells in COVID-19. Previous studies are mostly dependent on flow cytometry or bulk RNA sequencing (13, 14), but they generally fail to provide sensitivity to tiny distinctions in cells. As the technology develops, single-cell RNA sequencing analysis provides a powerful approach to obtain an unbiased and comprehensive visualization of the immunological profiles of PBMCs in patients with COVID-19. Compared with bulk RNA sequencing, it has a single-cell level resolution, which can estimate the whole changes of cell subsets, the immune cell function of individual cell, and the correlation between different cell subsets and cytokines.

In the present study, we reanalyzed the recently published single-cell RNA sequencing datasets of COVID-19 patients, and we confirmed decreased NKT cells in PBMCs of COVID-19 patients, which was associated with the severity and outcomes of the disease. The number of NKT cells in poor-outcome patients was relatively low, which is consistent with the results of previous studies (27). The decrease of circulating NKT cells may be caused by promoted NKT cell death, down-modulation of TCR in NKT cells, and NKT cells migration to the lung. Recent findings did not support the hypothesis of TCR down-modulation (14). Our finding also obtained no evidence of CD1d deficiency in COVID-19 (**Figure 7A**). Indeed, NKT cells in airways are undetectable, while a previous study concerning supernatants of endotracheal aspirates of COVID-19 patients found that the frequency of MAIT cells in airways was high (14). In addition, It is reported that the number of NKT cells in the lungs of patients with chronic obstructive pulmonary disease (COPD) is also increased (49). Our finding indicated that the number of circulating NKT cells in COVID-19 decreased, accompanied by changed functions of NKT cells such as promoted activation and increased cytokines secretion.

The decrease of circulating NKT cells can be recognized as an indicator to predict the prognosis of COVID-19 from recent researches (48). Our finding of single-cell RNA sequencing data analysis indicated that the proportion of Tim-3+ NKT cells in COVID-19 patients was higher than that in healthy controls, which was different from Tim-3- NKT cell subsets. Tim-3+ NKT cells increased in COVID-19, as the disease progressed. The increase of Tim-3+ NKT cells may be used as a new indicator for COVID-19 disease severity and outcome. Moreover, the number change of Tim-3+ NKT cells may contribute to the functional changes of NKT cells in COVID-19.

ACE2 is one of the most important receptors on the host cells mediating SARS-CoV-2 infection by binding to the spike protein (36). CD147 and CD26 have emerged recently as potential receptors for SARS-CoV-2. SARS-CoV-2 enter T cells, which do not express ACE2, by binding to CD147. At the same time, Meplazumab (an anti-CD147 antibody) can inhibit SARS-CoV-2 replication (37). CD26 is a main cellular entry for MERS-CoV (50), and recent structural studies predict that CD26 directly interacts with SARS-CoV-2 spike protein (38), while some contradictory results also have been reported (51). Our findings indicated Tim-3+ NKT cells have a trend to express more CD147 and CD26 in COVID-19 patients, which might suggest Tim-3+ NKT subset cells are highly susceptible to SARS-CoV-2 infection.

Our previous studies indicated that up-regulated expression of Tim-3 is associated with NKT cell apoptosis, NKT cell activation, and cytokines production in polymicrobial intra-abdominal infection (39). In the present study, we validate the notion. Tim-3+ NKT cells present a higher trend to apoptosis, and the increased proportion of Tim-3+ NKT cells could result in more apoptotic NKT cells caused to fewer circulating NKT cells in COVID-19 patients. In addition, increased cytokines production such as *IFN-γ*, *IL-4*, and *IL-10* were observed in Tim-3+ NKT cells, which indicated that Tim-3+ NKT cells showed the capacity of activation and secreting inflammatory cytokines. The over-activation of Tim-3+ NKT cells may involve in the early cytokine storm in COVID-19 by producing more IFN-γ, IL-4, and IL-10 (52). And the exhaustion of Tim-3+ NKT cells, characterized by high expression levels of PD-1, PD-L1, and IL-10, may lead to the immune paralysis of NKT cells as a long-term impact of COVID-19 (15). Recent studies also evidence that Tim-3 is more specific for T cells exhaustion than PD-1 in COVID-19 (23, 53). Thymosin alpha 1 used for reversion of T cells exhaustion can reduce the mortality of severe COVID-19, accomplished with a decrease of PD-1 and Tim-3 expression on T cells (54). Collectively, the up-regulated Tim-3 expression in NKT cells may be potentially responsible for functional changes and depletion of NKT cells in COVID-19.

Our recent work showed that IL-12 produced by stimulated DCs significantly promoted the expression of Tim-3 in NKT cells in mice model of severe bacterial infection (39). Previous studies have also demonstrated that IL-12 produced by DCs induces the activation of NKT cells *in vivo* and promotes the IFN-γ secretion of NKT cells (55–57). In the present study, the production of IL-12 produced by DCs/monocytes in COVID-19 patients is more than that in healthy controls, and the level of IL-12 in patients with mild/moderate COVID-19 is higher than that in patients with severe/critical COVID-19, which is consistent with the results of a recent study (58). Moreover, our findings indicated that the frequency of Tim-3+ NKT cells is positively correlated with the frequency of DCs/monocytes that secrete IL-12 in COVID-19 patients.

In summary, by analyzing the published single cell datasets, we found that circulating NKT cells in COVID-19 patients decreased. The up-regulated expression of Tim-3 in NKT cells is associated with potential SARS-CoV-2 spike protein binding receptors, cell apoptosis, activation and exhaustion, and cytokines secretion of NKT cells. IL-12-secreting DCs/monocytes may be responsible for induced up-regulation of Tim-3 in NKT cells during SARS-CoV-2 infection. Tim-3+ NKT cells subset may be a potential indicator for predicting the outcome in COVID-19 patients, and there is a possibility that the regulation of NKT cell by Tim-3 signal pathway could be a new strategy for elimination of lethal epidemic caused by SARS-CoV-2 infection. Further studies are needed to validate the findings.

## Data Availability Statement

The datasets presented in this study can be found in online repositories. The names of the repository/repositories and accession number(s) can be found in the article/**Supplementary Material**.

## Ethics Statement

Ethical review and approval were not required for the study on human participants in accordance with the local legislation and institutional requirements. Written informed consent for participation was not required for this study in accordance with the national legislation and the institutional requirements.

## Author Contributions

Z-HT designed and supervised the study, analyzed data, and wrote the manuscript. JY, TC, LT, HD, DC, JL, HW, TT, CZ, ZL, and LD analyzed data. XY analyzed data and contributed to writing the manuscript. All authors contributed to the article and approved the submitted version.

## Funding

This work was supported in part by National Natural Science Foundation of China 81873870 (Z-HT).

## Conflict of Interest

The authors declare that the research was conducted in the absence of any commercial or financial relationships that could be construed as a potential conflict of interest.

## Publisher’s Note

All claims expressed in this article are solely those of the authors and do not necessarily represent those of their affiliated organizations, or those of the publisher, the editors and the reviewers. Any product that may be evaluated in this article, or claim that may be made by its manufacturer, is not guaranteed or endorsed by the publisher.
