# Supplementary material for: Increased Expression of Tim-3 Is Associated With Depletion of NKT Cells In SARS-CoV-2 Infection
Source: Front Immunol. 2022 Feb 16;13:796682. doi: 10.3389/fimmu.2022.796682 (PMC8889099; doi:10.3389/fimmu.2022.796682)
Supplement: Supplementary file 1 [file DataSheet_1.docx]

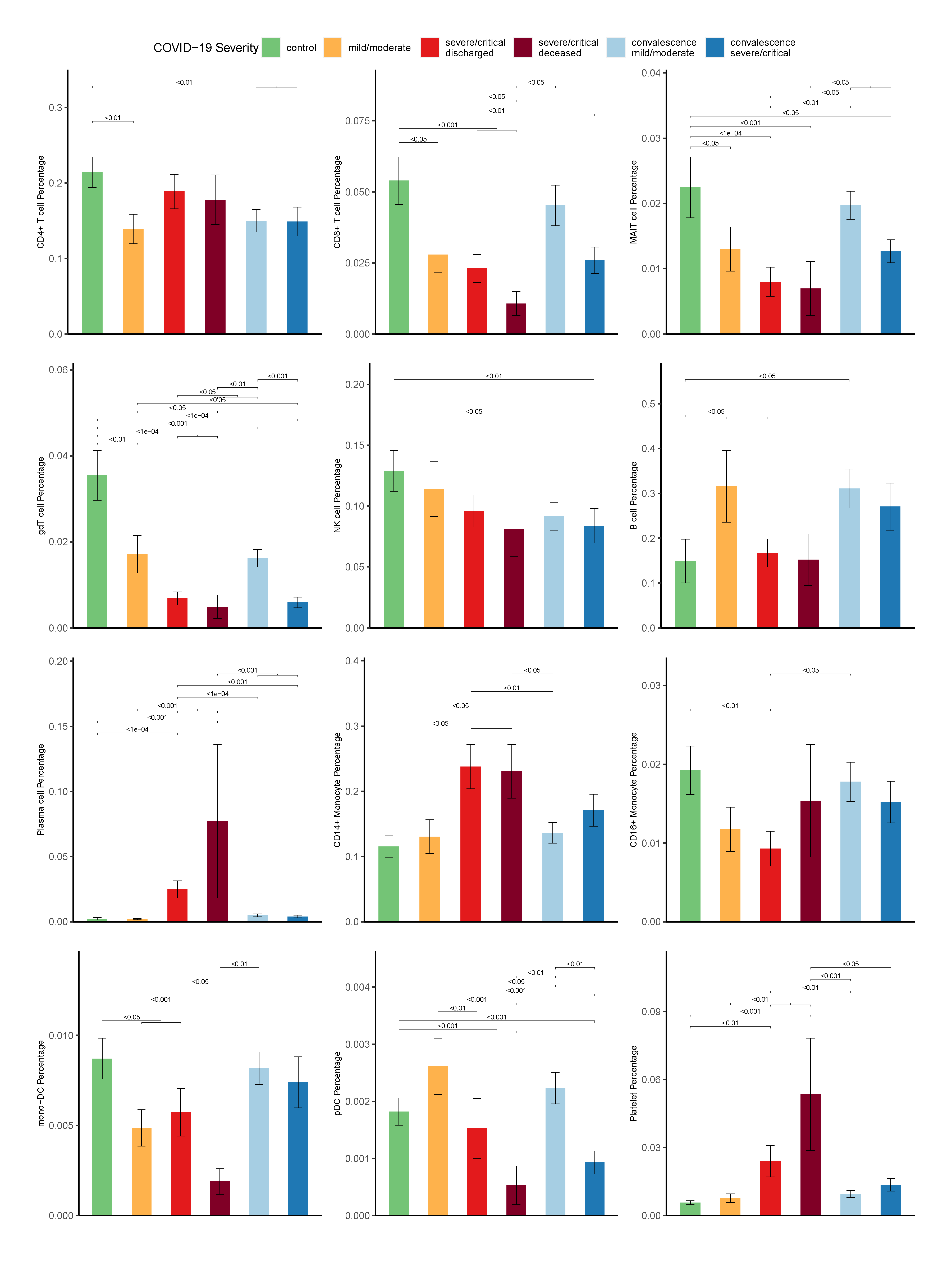


**Supplementary Figure S1.** Percentage of other PBMC cells in COVID-19 patients and controls derived by single cell datasets. CD4+ T cells (*CD3D*+*CD4*+), CD8+ T cells (*CD3D*+*CD8A*+), γδ T cells (*TRGV9*+*TRDV2*+), MAIT cells (*SLC4A10*+*TRAV1-2*+), NK cells (*KLRF1*+), B cells (*MS4A1*+), plasma B cells (*MZB1*+), CD14+ monocytes (*LYZ*+*CD14*+), CD16+ monocytes (*LYZ*+*FCGR3A*+), mono-DCs (*CD1C*+), pDCs (*LILRA4*+) and platelets (*PPBP*+). (Wilcoxon test)


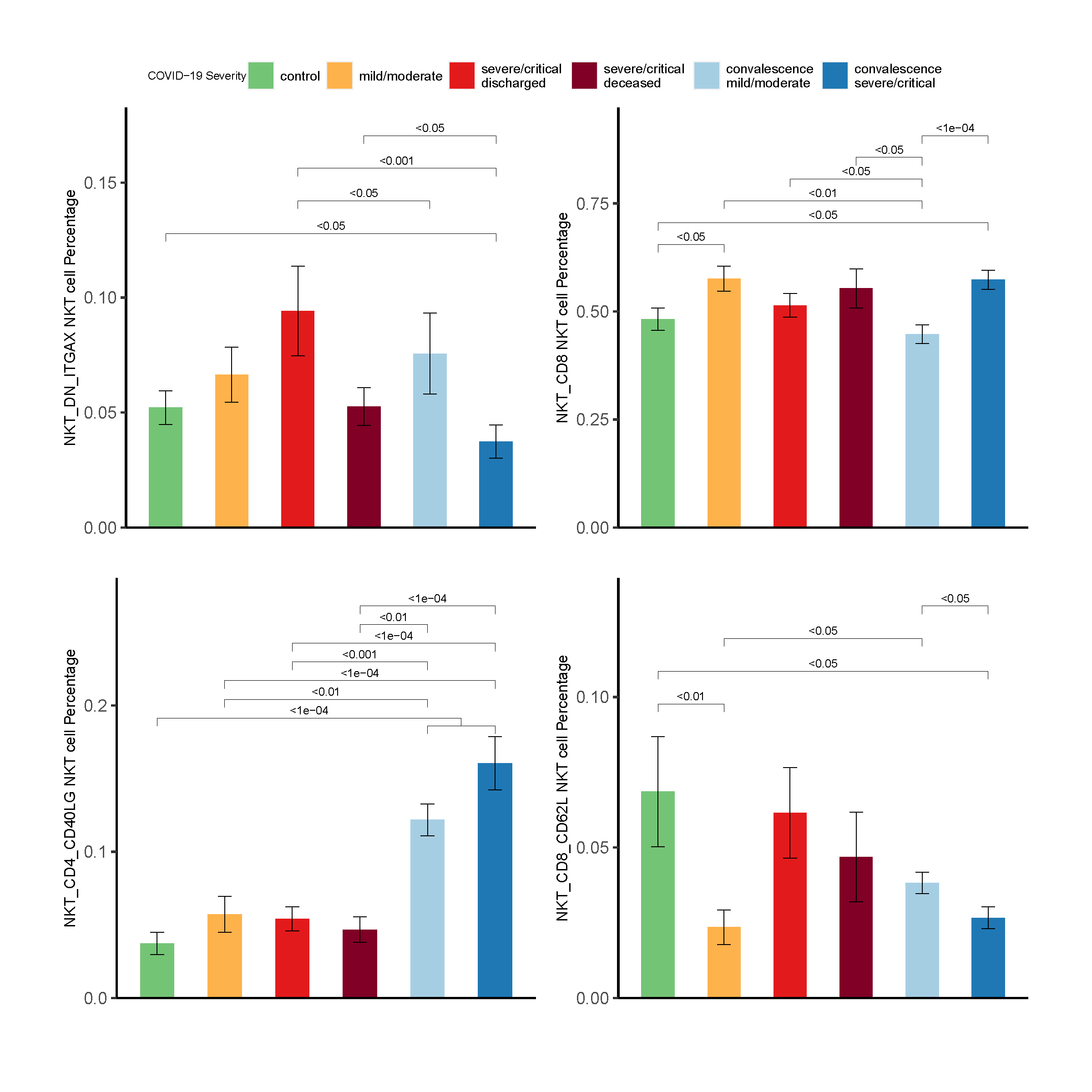


**Supplementary Figure S2.** Percentage of other NKT cell clusters in COVID-19 patients and controls. (Wilcoxon test)
